# Future directions on low-energy radiation dosimetry

**DOI:** 10.1038/s41598-021-90152-3

**Published:** 2021-05-19

**Authors:** G. Massillon-JL

**Affiliations:** Instituto de Física, Universidad Nacional Autónoma de México, 04510 Coyoacan Mexico City, Mexico

**Keywords:** Physics, Atomic and molecular physics, Atomic and molecular interactions with photons

## Abstract

For more than one century, low-energy (< 100 keV) photons (x-rays and gamma) have been widely used in different areas including biomedical research and medical applications such as mammography, fluoroscopy, general radiography, computed tomography, and brachytherapy treatment, amongst others. It has been demonstrated that most of the electrons produced by low photon energy beams have energies below 10 keV. However, the physical processes by which these low energy electrons interact with matter are not yet well understood. Besides, it is generally assumed that all the energy deposited within a dosimeter sensitive volume is transformed into a response. But such an assumption could be incorrect since part of the energy deposited might be used to create defects or damages at the molecular and atomic level. Consequently, the relationship between absorbed dose and dosimeter response can be mistaken. During the last few years, efforts have been made to identify models that allow to understand these interaction processes from a quantum mechanical point of view. Some approaches are based on electron-beam − solid-state-interaction models to calculate electron scattering cross-sections while others consider the density functional theory method to localize low energy electrons and evaluate the energy loss due to the creations of defects and damages in matter. The results obtained so far could be considered as a starting point. This paper presents some methodologies based on fundamental quantum mechanics which can be considered useful for dealing with low-energy interactions.

## Introduction

The utilization of ionizing radiation to diagnose and cure a variety of diseases is dated almost since the discovery of X-rays and radioactivity. But, the use of ionizing radiation always leads to a certain amount of energy deposited into the matter as a consequence of multiple physical phenomena such as elastic and inelastic collisions between charged particles (ions and/or electrons) and the electrons of the medium. Radiation dosimetry is the study of the energy deposition in matter by ionizing radiation and its quantification in terms of absorbed dose. However, in spite of many years of research, dosimetry is still challenging mainly if the radiation energy is low (below 100 keV). For instance, these energy levels include brachytherapy treatments^1^, superficial radiotherapy^2^, mammography^3^ or biomedical investigations^4–6^.

In the low energy region used in the clinic for diagnosis or treatment, many dosimetry studies have been performed using different kind of dosimeters or through Monte Carlo simulations^1–3,7,8^. However, the absorbed dose obtained is generally different from one dosimeter to another, caused possibly by secondary electron contamination^8^ and the high dose-rate gradient that exists at very short distances from the radiation sources. A large part of the problem is indeed the accurate determination of the absorbed dose^7,8^. Owing to the scarcity of fundamental research, it is not yet clear which physical processes are involved. Therefore, basic investigations are still needed to further improve the accuracy of the dosimetry and clinical application of low energy photon beams. This will require, the understanding of the interaction processes of low energy radiation with the materials used as dosimeters (device used to measure the absorbed dose) at the atomic and molecular level which would afford a better and safer use of ionizing radiation in medicine and consequently prevent any undesirable effect to the patients.

When photons interact with matter, the absorbed dose deposited at a certain point within its volume is obtained through the energy spectra of generated electrons which ionize the medium and the irradiated mass which can be evaluated through the continuous slowing down approximation (CSDA) range^9^. In addition, low-energy secondary electrons (SE), have been shown to be the main participants in the processes of ionization and responsible for radiation damage in matter^10^. Therefore, knowledge of the electron spectra^11,12^ and their corresponding CSDA range^13^ are essential to accurately determine the absorbed dose and evaluate the radiation effect in matter. For accurate low-energy dosimetry, there are two fundamental questions to ask: How many electrons are produced during the interaction? and is all the energy deposited within a dosimeter sensitive volume transformed into a response? Knowing the answer to these questions should give rise to a better relationship between absorbed dose and a dosimeter response.

The correct evaluation of the electron spectra strongly depends on how well the electron cross-section is known. The conventional method to calculate cross-sections for charged particle’s interaction with matter is based on the Bethe approximation^14^. Such an approximation uses the concept of mean excitation energy of the medium which is sensitive to the valence electron arrangement and depends on the electronic structure details of such medium^14^. Subsequently, the mean excitation energy can be considered valid only for particles with energies higher than the binding energy of the deepest inner shell of the atom. Thus, the Bethe approximation is not considered reliable in the low-energy region for electrons energies below 10 keV^14^. The most complete cross-section data reported in the literature are for electrons with energies greater than or equal to 1 keV [even though considered not accurate^14^] while the CSDA ranges have been provided for electrons with energies down to 10 keV^15^ due the uncertainty level.

A recent study^11^ based on the Bethe approximation about electron spectra generated in LiF and liquid water by several low-energy X-ray beams from 20 to 300 kV, ^137^Cs and ^60^Co has been reported. The contribution of secondary electrons (SE: produced by electron–electron interactions) relative to the total electron fluence (TEF: “primary electrons” generated directly by photons + “secondary electrons”) for LiF and liquid water, respectively has been evaluated. It was observed that independent of the media, SE generated by ^137^Cs and ^60^Co gamma with energies between 1 and 10 keV represent 60%-90% and 70%-90% of the TEF, respectively. Whereas for electrons below 10 keV produced by the low-energy X-rays, there is an increasing predominance of SE and depending on the photon energy beam, between 40 and 80% are expected to be below 1 keV.

In addition, several studies have revealed that the relative efficiency (*RE:* ratio of a dosimeter’s response per absorbed dose due to the radiation field of interest with respect to the same for ^60^Co gamma) of a dosimeter exposed to low photon energy strongly depends on how the absorbed dose is evaluated (experimental or Monte Carlo)^16,17^. In the case of radiochromic film’s response after exposure to low photon energies, differences up to 20% were observed on *RE* depending on how the absorbed dose was evaluated^16^. Similarly, a study of LiF:Mg,Ti exposed to low photon energy beams indicated that depending of the method used to evaluate the absorbed dose, discrepancies of 4%-18% on *RE* can be obtained^17^. Thus, to evaluate the impact of the electron range on the absorbed dose, the CSDA range for electrons with energy between 1 and 10 keV in LiF has been assessed^13^ using experimental data published by Morbitzer and Scharmann^18^. With the obtained results, the irradiated mass was estimated and a better agreement on *RE* of 0.7%-8% was observed despite of the uncertainties^13^. It was concluded that the absorbed dose delivered by low photon energy is not accurately known^13^.

On the other hand, outside of radiotherapy fields where low photon energy spectra exist^18,19^ discrepancies are commonly observed on the absorbed dose measured with different dosimeters^20–22^. This can be associated to two possible phenomena: a lack of information about the electron interaction at low energy and/or the limited understanding about the relationship between the absorbed dose and the dosimeter’s response.

The Linear energy transfer (LET) has been proposed by the international commission on radiation units and measurement^23^ as a non-stochastic quantity to describe the quality of an ionizing radiation beam. The track-average, ${L}_{\Delta ,T}$, and dose-average, ${L}_{\Delta ,D}$, LET are two quantities that have been used to quantify the ionizing radiation-induced effect or damage in biological and physical systems. ${L}_{\Delta ,T}$ represents the average energy lost by charged particles due to collisions in crossing a certain distance with energy transfers less than some specified energy cutoff value, $\Delta$, whereas ${L}_{\Delta ,D}$ is the average LET associated to the absorbed dose distribution. $L_{\Delta ,T}$ and $L_{\Delta ,D}$ are defined as follows

1 $$ L_{\Delta ,T} = \frac{{\mathop \smallint \nolimits_{\Delta }^{{E_{\max } }} L_{\Delta } \left( E \right)\Phi \left( E \right)dE + S\left( \Delta \right)\Phi \left( \Delta \right)\Delta }}{{\mathop \smallint \nolimits_{\Delta }^{{E_{\max } }} \Phi \left( E \right)dE + \Phi \left( \Delta \right)\Delta }}, $$

2 $$ L_{\Delta ,D} = \frac{{\mathop \smallint \nolimits_{\Delta }^{{E_{\max } }} L_{\Delta}^{2} \left( E \right)\Phi \left( E \right)dE + S^{2} \left( \Delta \right)\Phi \left( \Delta \right)\Delta }}{{\mathop \smallint \nolimits_{\Delta }^{{E_{\max } }} L_{\Delta } \left( E \right)\Phi \left( E \right)dE + S\left( \Delta \right)\Phi \left( \Delta \right)\Delta }}, $$

where $S\left( E \right)$, $L_{\Delta } \left( E \right)$, ${\Phi }\left( {\text{E}} \right)$ and $E$ represent the unrestricted and restricted electronic stopping power, the electron energy fluence, and the electron energy, respectively. The term $S(\Delta )\Phi (\Delta )\Delta$ is an approximation proposed by Nahum^24^ which considers that the electrons contributing to the fluence at energies below the energy cutoff value would have the same energy equal to the energy cutoff.

Note that knowledge about the electron fluences generated by photons is not only essential to evaluate accurately absorbed dose but also for a correct calculation of track and dose-average LET.

Cabrera-Santiago and Massillon-JL have investigated ${L}_{\Delta ,T}$ ^11^ and ${L}_{\Delta ,D}$ ^25^ of electrons generated in LiF and liquid water by low energy X-rays from 20 to 300 kV, ^137^Cs and ^60^Co gamma. Due to the fact that it is not possible to follow electrons with energies below 1 keV, they used the approximation proposed in Eqs. (1) and (2). It was concluded that such approximation might not be accurate and would not solve the problem of the incompleteness of the electron fluence generated by photons^11^.

Thus, considering that most of electrons generated by low photon energy beams have energies below 10 keV and the interaction process in that energy interval is not well known, one can argue that the accurate determination of the absorbed dose in low-energy radiation field is questionable. A quantum mechanics approach could offer a path to adequately forecast the low-energy radiation dosimetry’s future. The present article is concerned with possible methodologies considered promising for responding to the challenge in low-energy dosimetry.

## Research fields that will have a direct impact on low-energy dosimetry

### Low energy electron interaction models

Interaction processes of electron with energies below 1 keV have caught the attention of several groups^26–33^ due to the application of low energy radiation in different fields of research besides dosimetry like radiology, radiobiology, biomedical investigations, nanotechnology, and scanning electron microscopy. The material of dosimetric interest most studied in the sub-keV energy range is liquid water^26–30,34–37^ due to its application in radiobiological research.

For incident electrons with energies between 10 keV and the bandgap energy (~ 10 eV), valence electrons’ scattering is expected to be the most significant energy loss mechanism in low-*Z* materials like organic compounds similar to those used in dosimetry, while core electrons participate in less than 10% of the energy lost for interactions between 1 and 10 keV. Below the bandgap, phonon interactions might be important. But for the sake of this work, we will be concentred on electrons with energies down to the bandgap of the material.

The complex dielectric function provides a complete description of how a condensed medium responds to the perturbation of an external point charge as a group of interactions between electrons and atoms. Besides, it contains contributions from both valence and core electrons. Thus, several groups have calculated electron cross-sections in different media at the sub-keV region through the dielectric function model or other methods. Amongst the different groups, Emfietzoglou and colleagues have extensively studied differential cross-section interaction and mean free paths in liquid water for energy electrons down to 10 eV^28,38^, 50 eV^29^ and 100 eV^26^ using their own developed dielectric response model. Nevertheless, different approaches in the same model for the dielectric response function show remarkable differences on the cross-section obtained at energies below 200 eV^26,27,30^. Besides the GEANT Monte Carlo (MC) code that includes cross-sections for low energy electrons in liquid water, to the best of our knowledge, the PENELOPE is the only publicly available MC code that allow simulating electron energies down to 50 eV in materials other than liquid water, such as compounds. But, this 50 eV limit is doubtful since PENELOPE disregards aggregation effects considered critical for low-energy electron interactions in condensed matter by rescaling the mean free paths to the mass density of the medium and using interaction cross sections based on isolated atoms^31^. The Tanuma group has successfully calculated inelastic mean free paths (related inversely to the electron inelastic cross section, σ, via λ^-1^ = Nσ, N: density of scatterers) for a broad range of materials including elemental solids^39–42^ and compounds^43–47^ using the dielectric function model proposed by Penn^48^. The Penn model, which is valid for materials that have a known dielectric function, $\epsilon (q,\omega )$, uses experimental optical data (i.e. zero momentum transfer, q = 0) and the theoretical Lindhard dielectric function to calculate the inelastic scattering probability depending on the energy loss and momentum transfer. The most complete formula is the full Penn algorithm (FPA), which contemplates the extension of the optical data to nonzero momentum transfer (q $\ne$ 0) and requires triple integrations over the plasmon energy (*ω*_*p*_), the momentum transfer (*q*), and the energy loss (*ω*)^48^. The FPA is considered reliable at electron energies down to 50 eV. Below, a brief description of the method is presented.

#### The full Pen Algorithm (FPA)

Electromagnetic interaction between the charge and spin of an incident particle and those of atomic electrons can be sub-divided into two terms: the Coulomb interaction, which exerts a force parallel to the momentum transfer, *q*, called “longitudinal excitation,” and the interaction through virtual photons that are perpendicular to *q* named “transverse excitation”^49^. Thus, from a standpoint of quantum theory, the relativistic differential cross section (DCS) for inelastic scattering has a longitudinal component and a transverse one^49^. But, for electrons with energies less than 500 keV, the transverse component of DCS has been reported to be negligible^32^. Thus, for low-energy interactions, only the longitudinal excitation is considered and consequently the relativistic DCS is defined as^49^:

3 $$  \frac{{d^{2} \sigma_{L} }}{d\omega dq} = \frac{2}{{\pi Nv^{2} }}{\mathbf{Im}}\left[ {\frac{ - 1}{ \epsilon{\left( {q,\omega } \right)}}} \right]\frac{1}{q}, $$

where $N$ and $v$, are the number of atoms per volume unit and the incident electron’s velocity, respectively. $q$ represents the momentum transfer and $\omega$, the energy loss. ${\mathbf{Im}}\left[ {\frac{ - 1}{ \epsilon{\left( {q,\omega } \right)}}} \right]$ is the energy loss function (ELF) that characterises the inelastic scattering process. $\epsilon \left( {q,\omega } \right)$ is the complex dielectric function and related to the response of a solid to an external electromagnetic perturbation. Equation 3 considers the Hartree atomic units, ${m}_{e}=e=\hslash =4\pi {\varepsilon }_{0}=1$, where *e* is the elementary charge, and *ħ* the reduced Planck constant.

One of the important aspects of Eq. 3 is the integration limit over which the cross-section should be calculated considering all allowed $\omega$ and *q* values. Some groups have considered the indistinguishability between the incident electron and the scattered one by using a high energy approximation for non-conducting materials^26,36^.

**Figure 1:**
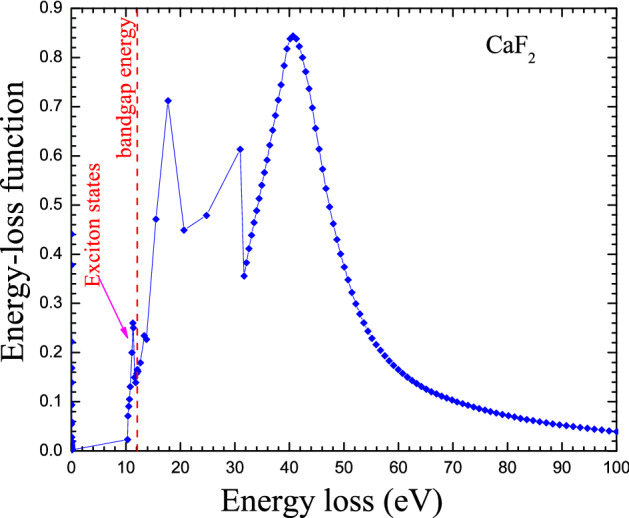
Energy loss function for CaF_2_ where the exciton states can be observed just below the bandgap energy.

But it is worth mentioning that, a free electron in the conduction band that leaves a hole in the valence band can be imagined as interacting through a Coulomb-like field. This would give rise to the possible existence of electron–hole bound states with excitation energies lower than the bandgap energy called excitons. Thus, the exciton effects are particularly important near the bandgap energy of insulators like those used in dosimetry as shown in Fig. 1. Figure 1 displays the energy-loss function (ELF) for CaF_2_ compound where some structures correspond to the exciton sates can be observed. The ELF curve is made from experimental optical data published in the literature^37^ and details about how to build this curve can be found in Ref.^37^.

Thus, in contrast to the indistinguishability between electrons, in a recent study for dosimetric materials, Flores-Mancera and colleagues^37^ have established an integration domain based on the bandgap energy (${E}_{\mathrm{g}}$)^50^, the valence band width (${w}_{\mathrm{VB}}$), and exciton interactions to which the lower integration limit (${\omega }_{\mathrm{min}}$) is associated (see Flores-Mancera et al*.* 2020). Thus, the resultant integral of Eq. 3 becomes

4 $$  \sigma_{L} = \frac{{\left( {1 + T^{\prime}/c^{2} } \right)^{2} }}{{1 + T^{\prime}/2c^{2} }}\frac{1}{{\pi N T^{\prime}}}\mathop \smallint \limits_{{\omega_{{{\text{min}}}} }}^{{T^{\prime} - w_{{{\text{VB}}}} }} \mathop \smallint \limits_{{q_{ - } }}^{{q_{ + } }} {\text{Im}}\left[ {\frac{ - 1}{\epsilon{\left( {q,\omega } \right)}}} \right]\frac{dq}{q}{\text{d}}\omega , $$

where

5 $$ q_{ \pm } = \sqrt {T^{\prime}\left( {2 + T^{\prime}/c^{2} } \right)} \pm \sqrt {\left( {T^{\prime} - \omega } \right)\left( {2 + \left( {T^{\prime} - \omega } \right)/c^{2} } \right) } $$

where $T^{\prime} = T - E_{{\text{g}}}$, T the difference between the energy of the incident electron and the energy at the bottom of the valence band.

To compute Eq. (4), the FPA proposes the following approach^40^:

6 $$  {\mathbf{Im}}\left[ {\frac{ - 1}{\epsilon{\left( {q,\omega } \right)}}} \right] = \mathop \smallint \limits_{0}^{\infty } d\omega_{p} G\left( {\omega_{p} } \right) {\mathbf{Im}}\left[ {\frac{ - 1}{{\epsilon_{L} \left( {q,\omega ;\omega_{p} } \right)}}} \right], $$

7 $$  G\left( {\omega_{p} } \right) = \frac{2}{{\pi \omega_{p} }}{\mathbf{Im}}\left[ {\frac{ - 1}{\epsilon{\left( {\omega_{p} } \right)}}} \right], $$

where $\epsilon_{L} \left( {q,\omega ;\omega_{p} } \right)$ is the Lindhard free electron dielectric function, $\omega_{p} = \sqrt {4\pi n}$ is the plasmon energy with electron density, *n,* and $\epsilon \left( {\omega_{p} } \right)$ is the optical dielectric function (i.e. $q = 0$).

Considering a free electron gas with a given plasmon frequency, ${\omega }_{p}$, there is a small area on the $\left(q,\omega \right)$-plane along the plasmon dispersion line where both the imaginary part of the dielectric function, ${\epsilon }_{L}^{i}\left(q,\omega ;{\omega }_{p}\right)=0$ and the real part, ${\epsilon }_{L}^{r}\left(q,\omega ;{\omega }_{p}\right)=0$ can contribute to the integration in Eq. (6). That is, the Lindhard energy loss function does not converge along that plasmon dispersion line, whereas only the single electron excitation is allowed when ${\epsilon }_{L}^{i}\left(q,\omega ;{\omega }_{p}\right)\ne 0$. Thus, according to the FPA, the ELF in Eq. 6 can be portrayed as a combination of the plasmon pole [i.e. ${\epsilon }_{L}^{i}\left(q,\omega ;{\omega }_{p}\right)=0]$ and the single-electron excitation [i.e. ${\epsilon }_{L}^{i}\left(q,\omega ;{\omega }_{p}\right)\ne 0]$ such as:

8 $$  {\mathbf{Im}}\left[ {\frac{ - 1}{\epsilon{\left( {q,\omega } \right)}}} \right] = {\mathbf{Im}}\left[ {\frac{ - 1}{\epsilon{\left( {q,\omega } \right)}}} \right]_{{{\text{pl}}}} + {\mathbf{Im}}\left[ {\frac{ - 1}{\epsilon{\left( {q,\omega } \right)}}} \right]_{{{\text{se}}}} $$

where pl and se refer to the plasmon pole and the single-electron, respectively^40^,

where

9 $$\text{Im} \left[ {\frac{{ - 1}}{\epsilon{\left( {q,\omega } \right)}}} \right]_{{pl}}  = G\left( {\omega _{0} } \right)\frac{\pi }{{\partial \epsilon_{L}^{r} \left( {q,\omega ;\omega _{p} } \right)/\partial \omega _{{p\omega _{p}  = \omega _{0} }} }}\theta \left( {q^{ - } \left( {\omega ;\omega _{0} } \right) - q} \right)  $$

and

10 $$  {\text{Im}}\left[ {\frac{ - 1}{\epsilon{\left( {q,\omega } \right)}}} \right]_{{{\text{se}}}} = \mathop \smallint \limits_{0}^{\infty } d\omega_{p} G\left( {\omega_{p} } \right){\text{ Im}}\left[ {\frac{ - 1}{{\epsilon_{L} \left( {q,\omega ;\omega_{p} } \right)}}} \right]\theta \left( {q^{ + } \left( {\omega ;\omega_{p} } \right) - q} \right)\theta \left( {q - q^{ - } \left( {\omega ;\omega_{p} } \right)} \right), $$

With

11 $$ q^{ \pm } \left( {\omega ;\omega_{0} } \right) = \pm k_{F} \left( {\omega_{p} } \right) + \sqrt {k_{F}^{2} \left( {\omega_{p} } \right) + 2\omega } , $$

12 $$ k_{F} = \left( {\frac{3\pi }{4}} \right)^{1/3} \left( {\omega_{p} } \right)^{2/3} $$

where $\omega_{0}$ is the numerical solution of the relation $\epsilon_{L}^{r} \left( {q,\omega ;\omega_{p} } \right) = 0$ when $\omega_{p} = \omega_{0} ,$ and $\theta \left( X \right)$ a step function. The first term of Eq. 11 corresponds to the incident wave vector of the particle before a collision and ${q}^{-}$ is the solution of the dispersion collision. Details about the solution of Eqs. (9) and (10) as well as the Lindhard dielectric functions can be found in Ref^41^.

**Figure 2:**
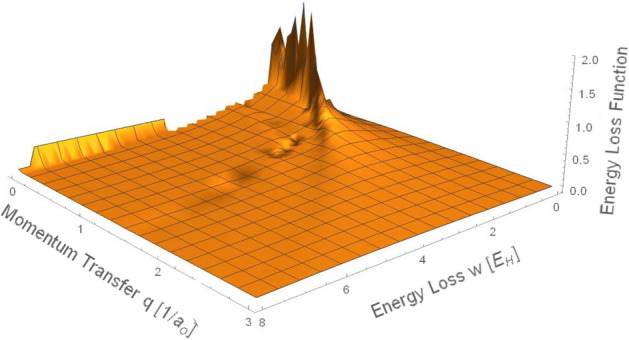
Perspective view of the energy loss function as a function of momentum transfer and energy loss calculated by the FPA for LiF.

For a given ω value, $q$ cannot surpass the Bethe ridge such as ${q}_{max}=\sqrt{2\omega }$ (in Hartree atomic units). Figure 2 presents the numerical solution of Eq. 8 after solving Eqs. (9) and (10). The optical data used to calculate the energy loss function were published previously by Flores-Mancera and colleagues^37^. Note that Fig. 2 only displays a perspective view of the energy loss function calculated by the FPA as a function of momentum transfer and energy loss for LiF^51^ in the limit of small ω and $q$ values. For large ω and $q$ values, the intensity of the energy loss function tends to zero. At very small energy loss values, the energy loss function for single electron excitation is more important than the contribution from the plasmon pole.

#### Importance of the FPA in dosimetry

Based on the FPA, a Monte Carlo (MC) code called Java Monte-Carlo Simulator for Secondary Electrons (JMONSEL) has been developed at the National Institute of Standards and Technology (NIST) to generate SE yield versus beam position (image) for a given sample shape and composition^33,52^. JMONSEL is based on an electron-beam − solid-state-interaction model which considers the interaction between the charge and spin of an incident particle and those of atomic electrons. The JMONSEL code has been used to interpret Scanning electron microscopy (SEM) dimensional measurements whose results were compared with measurements made using transmission electron microscopy and small-angle X-ray scattering. Comparisons between the simulation and the experiments show agreement at the subnanometer level^33^. To some extent, this agreement (see Fig. 7 in Ref.^53^) could be considered as a validation of the FPA method. Dosimetry and SEM have some similitude in the sense that in both cases the knowledge of SE yields is imperative. In SEM the SE yield is used to obtain an image while in dosimetry it is used to evaluate the absorbed dose. Thus, the JMONSEL approach could be considered adequate to study the electron cross-section in dosimetric materials.

**Figure 3:**
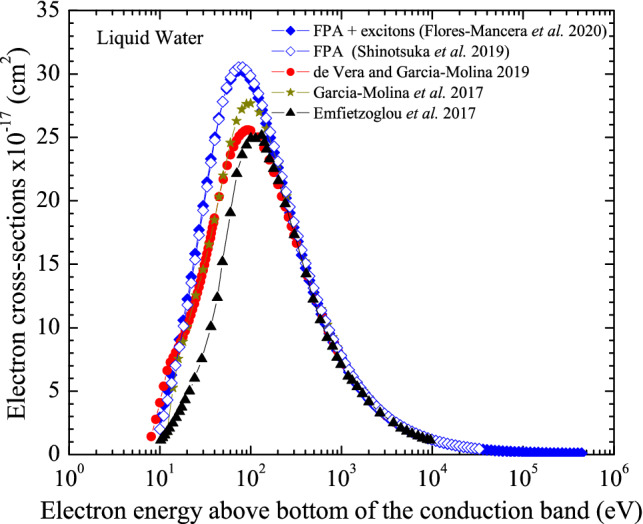
Electron cross-section for liquid water calculated with the FPA by Flores-Mancera et al*.* with exciton interactions included compared to data published by independent authors.

**Figure 4:**
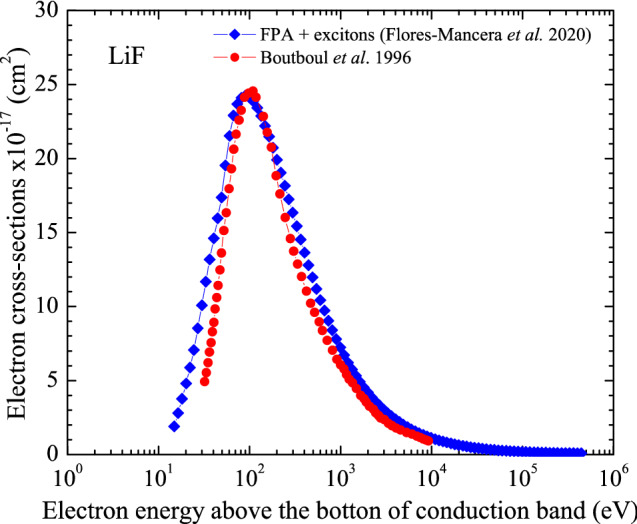
Electron cross-section for LiF calculated with the FPA by Flores-Mancera et al*.* with exciton interactions included compared to data published by independent authors.

Figures 3 and 4 present the electron cross-section for liquid water and LiF obtained through the FPA with exciton interactions included using Eq. 4^37^ Also included in Figs. 3 and 4 are data reported in the literature for other dielectric function model approaches or for the same FPA without the inclusion of the excitons.

As seen in Fig. 3, for liquid water at electron energies above 100 eV, there is a slight difference (~ 2.5%) between the result for the FPA with and without inclusion of exciton interactions while at energies below, this difference is up to 29%. So, exciton interactions should not be neglected on the electron cross-section calculation in the low energy range. Comparing with the other results reported in the literature for liquid water, agreement can be observed only at energies above 200 eV independent of the method used, while differences up to 42% can be seen at energies below 200 eV. Discussion about the possible interpretation of the substantial discrepancies observed between the FPA and previously published works can be found in Ref.^37^. With respect to data shown in Fig. 4 for LiF, it can be seen that the curve from Boutboul et al*.*^50^ is narrower than that reported by Flores-Mancera and colleagues^37^. Such difference could possibly be associated to the integration domain plus the inclusion of the exciton interaction since the lower limit in the Boutboul et al*.*^50^ study was set to zero. Thus, even though the cross-section data are not yet conclusive, but based on the level of agreement with experiments reported for the JMONSEL code, the cross-sections calculated by Flores-Mancera and colleagues^37^ could be considered reliable and a similar path might be followed for other dosimetric materials.

### Quantification of the energy deposited by low-energy electrons into a dosimeter

Generally, in dosimetry, all the energy deposited in the dosimeter is assumed to be transformed into a certain response. However, this might not be correct since there is always some energy loss due to interaction processes at the atomic and molecular levels and the response is a consequence of that interaction. But, the quantification of the exact energy deposited within a dosimeter’s sensitive volume is not an easy task either since the interaction process could be considered as a many-body problem.

Density functional theory (DFT) is considered as a useful and efficient tool for calculations of the electronic structure of atoms, molecules, and solids of complex structure^54^. DFT is based on the use of the electronic density, $\rho \left(r\right)$, instead of the many-body wave function, $\uppsi$, to solve the electronic structure problem so that from the Schrödinger equation we have:

13 $$ \hat{H}\Psi \left( r \right) = E\psi \left( r \right)   \Rightarrow   E\left( \rho  \right) = \langle \Psi \left( \rho  \right)|\hat{H}\left| {\Psi \left( \rho  \right)} \right\rangle,$$

The total energy of a system consisting of *N* electrons can be written in terms of the electron density as:

14 $$ \rho \left( r \right) = \mathop \sum \limits_{i = 1}^{N} \left| {{\uppsi }_{i} \left( r \right)} \right|^{2} , $$

with ${\uppsi }_{i}$ the single particle wave functions.

Thus, the ground-state energy of an interacting inhomogeneous electron gas in a static potential, $V_{ext} \left( r \right)$, can be written in terms of the electronic density as^55^:

15 $$ E\left( \rho  \right) = T\left( \rho  \right) + \int {V_{{ext}} \left( r \right)\rho \left( r \right)dr + \frac{1}{2}} \iint {\frac{{\rho \left( {r,r^{\prime } } \right)}}{{\left| {r - r^{\prime } } \right|}}drdr^{\prime }  + E_{{xc}} \left( \rho  \right)},$$

where $T\left( \rho \right)$ is the kinetic energy of non-interacting electrons with density, $\rho \left( r \right)$. The second and third terms are the nuclear interaction energy and the classical Coulomb self-energy, respectively. $E_{xc} \left( \rho \right)$ represents the density functional exchange–correlation energy of an interacting system.

The success or failure of a functional theory strongly depends on how ${E}_{xc}\left(\rho \right)$ is described. To study solid systems like those used in dosimetry, the best approach is the hybrid functional density theory (H-DFT) which mixes non-local Hartree–Fock exchange (HFX) with semi-local DFT/generalized gradient approximation (GGA) exchange. The Exchange–correlation energy for hybrid functional is given by^56^:

16 $$ E_{xc} \left( \rho \right) = \alpha E_{x}^{HFX} \left[ {\left\{ {\psi_{i} } \right\}} \right] + \left( {1 - \alpha } \right)E_{x}^{DFT} \left[ \rho \right] + E_{c}^{DFT} \left[ \rho \right] $$

where $\alpha$ represents the fraction of HFX, and *E*x and *E*c are the density functionals for exchange and correlation, respectively. $E_{x}^{HFX} \left[ {\left\{ {\psi_{i} } \right\}} \right]$ is the Hartree–Fock exchange energy which can be expressed in terms of a density matrix and two-electron integrals as^56^:

17 $$ E_{x}^{HFX} \left[ {\left\{ {\psi_{i} } \right\}} \right] = - \frac{1}{2}\mathop \sum \limits_{\lambda \sigma \mu \nu } P^{\mu \sigma } P^{\lambda \nu } {\iint }\phi_{\mu } \left( {r_{1} } \right)\phi_{\nu } \left( {r_{1} } \right)\frac{1}{{r_{12} }}\phi_{\lambda } \left( {r_{2} } \right)\phi_{\sigma } \left( {r_{2} } \right)dr_{1} dr_{2} $$

where $P^{\nu }$ is the density matrix elements, $\left\{ {\phi_{\mu } \left( r \right)} \right\}$ is the atomic centered basis set, and the term ${\iint }\phi _{\mu } \left( {r_{1} } \right)\phi _{\nu } \left( {r_{1} } \right)\frac{1}{{r_{{12}} }}\phi _{\lambda } \left( {r_{2} } \right)\phi _{\sigma } \left( {r_{2} } \right)dr_{1} dr_{2}$ represents the four-centre two-electron repulsion integrals.

H-DFT is computationally demanding and expensive. But nowadays thanks to the existence of high-performance computers (HPC), it is possible to simulate systems with up to thousands of atoms.

**Figure 5:**
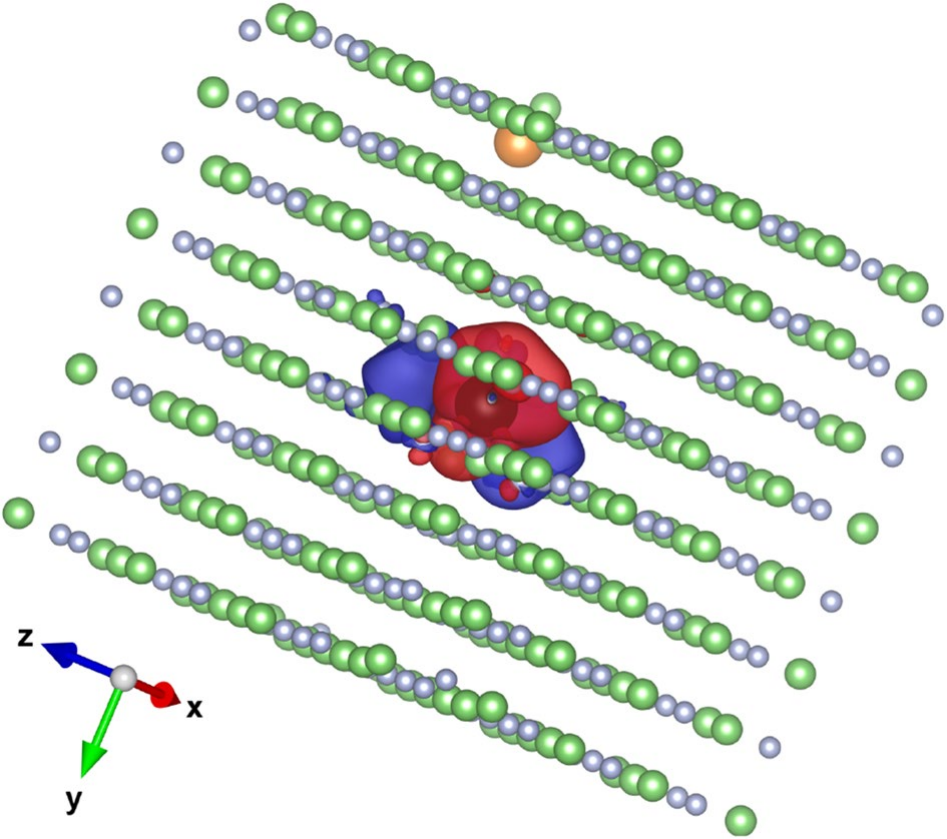
Spin density isosurface of LiF:Mg,Ti replicated into a 4 × 4 × 4 supercell containing 514 atoms. The grey, green, orange and black symbols represent the F, Li, Mg and Ti atoms, respectively. The red ( +) and blue (−) surfaces (value of 0.00134 e^−3^ Å each) are spin densities centred at the Ti *3d*-states.

Recently, first-principles H-DFT studies of defects and excess electrons in the dosimetric material LiF:Mg,Ti have been conducted to investigate the effects induced by ionizing radiation in matter at low energies^57^. In particular, the defect formation energies have been calculated. Figure 5 shows Spin density isosurface for LiF:Mg,Ti replicated into a 4 × 4 × 4 supercell containing 514 atoms. The results show that most of the defect states within the dosimeter’s sensitive volume which act as traps for electrons (see Fig. 5) are created by ionizing radiation^57^. And for a given defect created, a certain amount of energy is spent on its formation (see Table 2 in Massillon-JL *et al*^57^). This means that not all the energy absorbed into the dosimeter sensitive volume is transformed into a response. For example, about 10.33 eV are necessary to form an F-center which is the most common defect created by ionizing radiation in LiF. Besides, it has been found that the Mg defect in LiF:Mg,Ti generates an electronic structure similar to a void that acts as an electron trap and requires 15.34 eV to be created^57^. So, the total amount of energy consumed would depend on how many defects are created and their characteristics. This, of course, would be a function of the material in question. Thus, the energy spent for creating defects or other type of effect, like atom displacements and color centers for instance, should be subtracted from the energy deposited into the dosimeter volume in order to better establish the absorbed dose relationship with the dosimeter’s response. However, to be able to quantify that energy, more extensive research has to be done not only for LiF:Mg,Ti but also for several other dosimetric materials. In that case, the H-DFT could be a good startpoint.

## Conclusion

This paper has presented the state-of-the-art in low-energy radiation dosimetry and the existing challenges. For low-energy electron cross-sections which are not widely available for most compounds used as dosimeters, evidence has shown that the use of an electron-beam − solid-state-interaction model, in conjunction with some assumptions, could be very helpful. This will allow calculating electron yields more precisely which, in consequence, will have a direct impact on the accuracy of the absorbed dose determination. With respect to the relationship between the energy deposited and the response of a dosimeter, the hybrid-functional density theory (H-DFT) has demonstrated to be a promising tool for localizing secondary electrons within a dosimeter volume and for calculating the energy spent on creating defects or colors centers, etc. Afterwards, the amount of energy that can be truly transformed into the response of a dosimeter after exposure to ionizing radiation would be determinated more adequately.

## References

[1] Massillon JLG, Minniti R, Mitch MG, Soares CG, Hearn RA (2011). High-resolution 3D dose distribution measured for two low-energy x-ray brachytherapy seeds: ^125^I and ^103^Pd. Radiat. Meas..

[2] Schneider F, Clausen S, Thölking J, Wenz F, Abo-Madyan Y (2014). A novel approach for superficial intraoperative radiotherapy (IORT) using a 50 kV X-ray source: a technical and case report. J. Appl. Clinical Med. Phys..

[3] Palma BA, Rosado-Méndez I, Villaseñor Y, Brandan ME (2010). Phantom study to evaluate contrast-medium-enhanced digital subtraction mammography with a full-field indirect-detection system. Med. Phys..

[4] Frankenberg D, Kelnhofer K, Bär K, Frankenberg-Schwager M (2002). Enhanced neoplastic transformation by mammography X rays relative to 200 kVp X rays: Indication for a strong dependence on photon energy of the RBE_M_ for various end points *Radiat*. Res..

[5] Kellerer AM (2002). Electron Spectra and the RBE of X Rays. Radiat. Res..

[6] Göggelmann W, Jacobsen C, Panzer W, Walsh L, Roos H, Schmid E (2003). Re-evaluation of the RBE of 29 kV x-rays (mammography x-rays) relative to 220 kV x-rays using neoplastic transformation of human CGL1-hybrid cells. Radiat. Environ. Biophys..

[7] Hill R, Healy B, Holloway L, Kuncic Z, Thwaites D, Baldock C (2014). Advances in kilovoltage x-ray beam dosimetry. Phys. Med. Biol..

[8] Klevenhagen SC, D’Souza D, Bonnefoux I (1991). Complications in low energy x-ray dosimetry caused by electron contamination. Phys. Med. Biol..

[9] ICRU 90 Key Data for Ionizing-Radiation Dosimetry: Measurement Standards and Applications (Oxford University Press: International Commission on Radiation Unit and Measurements 2016)

[10] Martin F, Burrow PD, Cai Z, Cloutier P, Hunting D, Sanche L (2004). DNA strand breaks induced by 0–4 eV electrons: the role of shape resonances. Phys. Rev. Lett..

[11] Cabrera-Santiago A, Massillon-JL G (2016). Track-average LET of secondary electrons generated in LiF:Mg, Ti and liquid water by 20–300 kV x-ray, ^13^7Cs and ^6^0Co beams. Phys. Med. Biol..

[12] Massillon-JL G (2020). Track and dose-average LET dependence of Gafchromic EBT3 and MD-V3 films exposed to low-energy photons. Sci. Rep..

[13] Cabrera-Santiago A, Massillon-JL G (2016). Secondary electron fluence generated in LiF:Mg, Ti by low-energy photons and its contribution to the absorbed dose. AIP Conf. Proc..

[14] Seltzer SM, Berger MJ (1982). Evaluation of the collision stopping power of elements and compounds for electrons and positrons. Int. J. AppL Radiat. Isot..

[15] NIST ESTAR: Stopping Powers and Ranges for Electrons, disponible at https://physics.nist.gov/PhysRefData/Star/Text/ESTAR.html (2021)

[16] Massillon JLG, Cabrera-Santiago A (2019). Xicohténcatl-Hernández N. Relative efficiency of Gafchromic EBT3 and MD-V3 films exposed to low- energy photons and its influence on the energy dependence. Physica Med..

[17] Massillon-JL G, Cabrera-Santiago A, Minniti R, O’Brien M, Soares C (2014). Influence of phantom materials on the energy dependence of LiF:Mg, Ti thermoluminescence dosimeters exposed to 20–300 kV narrow x-ray spectra, ^13^7Cs and ^6^0Co photons. Phys. Med. Biol..

[18] Morbitzer L, Scharmann A (1964). Messung der Eindringtiefe von Elektronen und Ionen in dünnen Aufdampfschichten. Z. Phys..

[19] Jang S, Helen-Liu H, Mohan R (2007). Variations in energy spectra and water-to- material stopping-power ratios in three-dimensional conformal and intensity- modulated fields. Med. Phys..

[20] Scarboro SB, Followill DS, Howell RM, Kry SF (2011). Variations in photon energy spectra of a 6 MV beam and their impact on TLD response. Med. Phys..

[21] Bordy JM, Bessieres I, d’Agostino E (2013). Radiotherapy out-of-field dosimetry: Experimental and computational results for photons in a water tank. Radiat. Meas..

[22] Edwards CR, Mountford PJ (2004). Near surface photon energy spectra outside a 6 MV field edge. Phys. Med. Biol..

[23] Kry SF, Bednarz B, Howell RM, Dauer L, Followill D, Klein E, Paganetti H, Wang B, Wuu C-S, Xu XG (2017). AAPM TG 158: Measurement and calculation of doses outside the treated volume from external-beam radiation therapy. Med. Phys..

[24] ICRU 16 Linear Energy Transfer (Washington, DC: International Commission on Radiation Unit and Measurements 1970)

[25] Nahum AE (1978). Water/air mass stopping power ratios for megavoltage photon and electron beams. Phys. Med. Biol..

[26] Massillon JLG, Cabrera-Santiago A (2020). Dose-average linear energy transfer of electrons released in liquid water and LiF:Mg, Ti by low-energy x-rays, 137Cs and 60Co gamma. Biomed. Phys. Eng. Exp..

[27] Emfietzoglou D, Nikjoo H (2007). Accurate electron inelastic cross sections and stopping powers for liquid water over the 0.1–10 keV range based on an improved dielectric description of the Bethe surface. Radiat. Res..

[28] Kyriakou I, Incerti S, Francis Z (2015). 2015 Technical note: improve- ments in GEANT4 energy-loss model and the effect on low-energy electron transport in liquid water. Med. Phys..

[29] Emfietzoglou D, Moscovitch M (2002). Inelastic collision characteristics of electrons in liquid water. Nucl. Instrum. Methods. Phys. Res. B.

[30] Emfietzoglou D, Karava K, Papamichael G, Moscovitch M (2003). Monte Carlo simulation of the energy loss of low-energy electrons in liquid water. Phys. Med. Biol..

[31] Emfietzoglou D, Kyriakou I, Garcia-molina R, Abril I (2017). Inelastic mean free path of low-energy electrons in condensed media: beyond the standard models. Surf. Interface Anal..

[32] Fernańdez-Varea JM, Gonzaĺez-Muñoz G, Galassi ME, Wiklund K, Lind BK, Ahnesjö A, Tilly N (2011). Limitations (and merits) of PENELOPE as a track-structure code. Int. J. Radiat. Biol..

[33] Fernańdez-Varea JM, Salvat F, Dingfelder M, Liljequist D (2005). A relativistic optical-data model for inelastic scattering of electrons and positrons in condensed matter. Nucl. Instrum. Methods. Phys. Res. B.

[34] Villarrubia JS, Vladaŕ AE, Ming B, Kline RJ, Sunday DF, Chawla JS, List S (2015). Scanning electron microscope measurement of width and shape of 10 nm patterned lines using a JMONSEL- modeled library. Ultramicroscopy.

[35] Garcia-Molina R, Abril I, Kyriakou I, Emfietzoglou D (2017). 2017 Inelastic scattering and energy loss of swift electron beams in biologically relevant materials. Surf Interface Anal..

[36] Nguyen-Truong HT (2018). Low-energy electron inelastic mean free paths for liquid water. J. Phys. Condens. Matter..

[37] de Vera P, Garcia-Molina R (2019). Electron inelastic mean free paths in condensed matter down to a few electronvolts. J. Phys. Chem. C..

[38] Flores-Mancera MA, Villarrubia JS, Massillon JLG (2020). Electron inelastic mean free paths for LiF, CaF_2_, Al_2_O_3_, and liquid water from 433 keV down to the energy gap. ACS Omega.

[39] Emfietzoglou D, Kyriakou I, Abril I, Garcia-Molina R, Nikjoo H (2012). Inelastic scattering of low-energy electrons in liquid water computed from optical-data models of the Bethe surface. Int. J. Radiat. Biol..

[40] Shinotsuka H, Tanuma S, Powell CJ, Penn DR (2012). Calculations of electron stopping powers for 41 elemental solids over the 50 eV to 30 keV range with the full Penn algorithm. Nucl. Instrum. Meth. B.

[41] Shinotsuka H, Tanuma S, Powell CJ, Penn DR (2015). Calculations of electron inelastic mean free paths. X. Data for 41 elemental solids over the 50 eV to 200 keV range with the relativistic full Penn algorithm. Surf. Interface Anal..

[42] Tanuma S, Powell CJ, Penn DR (1988). Calculations of electron inelastic mean free paths for 31 materials. Surf. Interface Anal..

[43] Tanuma S, Powell CJ, Penn DR (2011). Calculations of electron inelastic mean free paths. IX. Data for 41 elemental solids over the 50 eV to 30 keV range. Surf. Interface Anal..

[44] Tanuma S, Powell CJ, Penn DR (1991). Calculations of electron inelastic mean free paths III data for 15 inorganic compounds over the 50–2000 eV range. Surf. Interface Anal..

[45] Tanuma S, Powell CJ, Penn DR (1994). Calculations of electron inelastic mean free paths V Data for 14 organic compounds over the 50–2000 eV range. Surf. Interface Anal..

[46] Shinotsuka H, Da B, Tanuma S, Yoshikawa H, Powell CJ, Penn DR (2017). Calculations of electron inelastic mean free paths. XI. Data for liquid water for energies from 50 eV to 30 keV. Surf. Interface Anal..

[47] Shinotsuka H, Tanuma S, Powell CJ, Penn DR (2019). Calculations of electron inelastic mean free paths. XII. Data for 42 inorganic compounds over the 50 eV to 200 keV range with the full Penn algorithm. Surf. Interface Anal..

[48] Penn DR (1987). Electron mean-free-path calculations using a model dielectric function. Phys. Rev. B.

[49] Fano U (1963). Penetration of protons, alpha particles, and mesons. Ann. Rev. Nucl. Sci..

[50] Boutboul T, Akkerman A, Breskin A, Chechik R (1996). Electron inelastic mean free path and stopping power modelling in alkali halides in the 50 eV−10 keV energy range. J. Appl. Phys..

[51] Castillo-Rico L. R. private communication (2021)

[52] Villarrubia JS, Ding ZJ (2009). Sensitivity of scanning electron microscope width measurements to model assumptions. J. Micro/Nanolithogr. Mems Moems.

[53] Vladár AE, Cizmar P, Villarrubia JS, Postek MT (2012). Can We Get 3D CD Metrology Right?. Proc. SPIE.

[54] Kohanoff J. Electronic structure calculations for solids and molecules: theory and computational methods (Cambridge University Press, 2006)

[55] Kohn W, Sham LJ (1965). Self-consistent equations including exchange and correlation effects. Phys. Rev..

[56] Guidon M, Hutter J, VandeVondele J (2010). Auxiliary density matrix methods for hartree-fock exchange calculations. J. Chem. Theory Comput..

[57] Massillon JLG, Johnston CSN, Kohanoff J (2019). On the role of magnesium in a LiF:Mg, Ti thermoluminescent dosimeter. J. Phys. Condens. Matter.

